# Household economic costs of norovirus gastroenteritis in two community cohorts in Peru, 2012–2019

**DOI:** 10.1371/journal.pgph.0002748

**Published:** 2024-07-10

**Authors:** Joan Neyra, Anita K. Kambhampati, Laura E. Calderwood, Candice Romero, Giselle Soto, Wesley R. Campbell, Yeny O. Tinoco, Aron J. Hall, Ismael R. Ortega-Sanchez, Sara A. Mirza

**Affiliations:** 1 U.S. Naval Medical Research Unit SOUTH, Lima, Peru; 2 National Center for Immunization and Respiratory Diseases, Centers for Disease Control and Prevention, Atlanta, Georgia, United States of America; 3 Cherokee Nation Operational Solutions, Tulsa, Oklahoma, United States of America; 4 Vysnova Partners, LLC, Greater Landover, Maryland, United States of America; 5 Division of Infectious Diseases, Walter Reed National Military Medical Center, Bethesda, Maryland, United States of America; University of the Western Cape, SOUTH AFRICA

## Abstract

While costs of norovirus acute gastroenteritis (AGE) to healthcare systems have been estimated, out-of-pocket and indirect costs incurred by households are not well documented in community settings, particularly in developing countries. We conducted active surveillance for AGE in two communities in Peru: Puerto Maldonado (October 2012–August 2015) and San Jeronimo (April 2015–April 2019). Norovirus AGE events with PCR-positive stool specimens were included. Data collected in follow-up interviews included event-related medical resource utilization, associated out-of-pocket costs, and indirect costs. There were 330 norovirus-associated AGE events among 3,438 participants from 685 households. Approximately 49% of norovirus events occurred among children <5 years of age and total cost to the household per episode was highest in this age group. Norovirus events cost a median of US $2.95 (IQR $1.04–7.85) in out-of-pocket costs and $12.58 (IQR $6.39–25.16) in indirect costs. Medication expenses accounted for 53% of out-of-pocket costs, and productivity losses accounted for 59% of the total financial burden on households. The frequency and associated costs of norovirus events to households in Peruvian communities support the need for prevention strategies including vaccines. Norovirus interventions targeting children <5 years of age and their households may have the greatest economic benefit.

## Introduction

Norovirus is a leading gastroenteric pathogen, associated with about one-fifth of acute gastroenteritis (AGE) cases worldwide [1]. The highest burden of illness is experienced by children in the first two years of life; however, norovirus is a common cause of AGE among all ages [2,3]. Clinical characteristics of norovirus infection are well described; norovirus has an incubation period of 12–48 hours, after which infected persons experience AGE symptoms including diarrhea, vomiting, and nausea lasting on average two to three days [4]. Though norovirus-associated AGE is typically self-limiting, severe disease is possible, affecting primarily children aged <5 years, older adults, and immunocompromised patients [3,5,6]. Further, due to its low infectious dose and high levels of shedding [7,8], outbreaks of norovirus-associated AGE spread quickly. This can cause widespread illness in households, as well as larger congregate settings such as long-term care facilities and universities, and affect operational readiness of groups such as military units [9–11].

The global economic burden of norovirus illness across all ages is estimated at approximately $60.3 billion annually, with productivity losses accounting for 93% of the total burden [12]. The Americas have been identified as the region with the highest norovirus-associated costs (health system and societal) [12,13]. While these studies provide valuable estimates of the economic costs of norovirus, results are based on models without individual economic data for laboratory-confirmed cases. Surveillance for individual norovirus cases is not systematically performed in most countries, and diagnostic testing for norovirus is relatively uncommon, so prevalence estimates and subsequent economic burden assessments typically rely on cross-sectional studies or healthcare data from developed countries. These studies do not typically collect costs accrued by symptomatic cases who do not require medical care, yet these costs could still have a sizable impact on individual household income and collectively on society.

Understanding the economic burden of norovirus in different regions, settings, and age groups is critical for assessing the potential economic benefits of norovirus interventions, including vaccines. Norovirus vaccines are currently under development and could be an effective method to reduce both disease burden and associated medical resource utilization and costs [14,15]. Our study aimed to estimate the financial burden of laboratory-confirmed norovirus-associated AGE on affected participants and their households, across all levels of care. We aimed to assess the out-of-pocket costs, including out-of-pocket medical and logistical expenses and the cost of self-treatment, as well as indirect costs related to lost wages and productivity among participants who experienced AGE events and their caregivers.

## Materials and methods

### Ethics statement

The study protocol (NAMRU6-2012-0013) was approved by the United States Naval Medical Research Unit SOUTH (formerly known as the United States Naval Medical Research Unit Number 6 [NAMRU-6]) Institutional Review Board in compliance with all applicable Federal regulations governing the protection of human subjects. Consent for participation was obtained from persons ≥18 years of age. Parental/guardian consent was obtained for participants <18 years of age, as well as assent from participants aged 10–17 years.

### Study population and design

Our data come from active surveillance cohort studies conducted in two peri-urban sites in geographically and ecologically distinct regions of Peru: Puerto Maldonado (PM), located in the department of Madre de Dios, and San Jeronimo (SJ), located in the department of Cusco. Both sites sit in the eastern part of Peru, which is relatively less wealthy than the country as a whole [16]. Of the two departments, Cusco is more developed; however, our study site in San Jeronimo, outside of the tourist center of Cusco, was slightly more rural and lower income compared to our site in the port town of Puerto Maldonado.

Households in these two peri-urban areas were randomly selected for enrollment from a previous census list; selection methodology has been previously described [17,18]. Briefly, households with at least 60% of members agreeing to participate were enrolled in an open cohort and were under surveillance from October 2012–August 2015 in PM and from April 2015–April 2019 in SJ. Demographic and household characteristics including monthly income were collected at enrollment and updated yearly. During the surveillance period, each household was visited two to three times per week to screen household members of all ages for AGE signs and symptoms.

An AGE event was defined as any of the following symptoms observed in a 24-hour period: 1) ≥3 loose or liquid stools, 2) ≥2 vomiting episodes or 3) ≥1 vomiting episode and ≥1 loose or liquid stool. New events were defined by onset of AGE symptoms after ≥2 weeks with no diarrhea or vomiting. Ill persons were asked to provide one stool sample per AGE event. We tested stool samples for norovirus GI and GII using duplex genogroup-specific real-time reverse transcription polymerase chain reaction (RT-qPCR) as previously described [17]. For this analysis, we included data from AGE events with a norovirus-positive stool specimen (hereafter referred to as norovirus events).

### Data collection

We conducted five follow-up visits during the 15 days after symptom onset for each norovirus event to collect information regarding signs and symptoms, healthcare-seeking behaviors, and household costs associated with the illness. The total cost of norovirus events included out-of-pocket and indirect costs. Out-of-pocket costs were defined as costs related to medical care and associated non-medical expenses incurred by patients or caregivers during the norovirus event. Medical care costs included payments for diagnosis and treatment (i.e., medications), cost of healthcare consultations, and expenses related to informal health providers such as traditional healers and pharmacy consultations. Associated non-medical expenses include transportation, lodging, phone calls, and meals purchased outside the home while seeking healthcare. Out-of-pocket costs were collected in Peruvian soles and converted to United States dollars (USD, $) using the official exchange rate per month and year published by Banco Central de Reserva de Perú (BCRP). Once in USD, costs were adjusted for inflation to December 2019 using the Gross Domestic Product: Implicit Deflator [19].

Indirect costs were those related to productivity losses of the ill person or caregiver from absence or reduced economic activities due to the norovirus event. Productivity losses were evaluated using a previously published daily index and were calculated as total days lost from paid work or unpaid activities (e.g., household work or school) [20]. We assigned a value of 0.25 to each day that the participant conducted routine activities but had symptoms, 0.5 to each partially absent day, and 1 to each day of total absence.

To assess the indirect cost associated with productivity losses from norovirus illnesses, we used a combined approach to include both individuals with paid employment, as well as those whose work was not remunerated with money. For ill persons and their caregivers that reported missed income, we used the total reported value throughout the follow-up period. For those that reported lost time, but no associated monetary value (e.g., when the ill person or their caregiver was a homemaker or student), we imputed an economic value for the lost time for working age individuals ≥15 years of age; we used the Peruvian annual GDP per capita as a proxy, prorated to the day and then multiplied it by the number of days missed from work due to norovirus illness. Indirect costs were also adjusted for inflation up to December 2019 as described above. We then summed the total values for participants and caregivers to estimate the total indirect cost of each norovirus event.

### Statistical analysis

Out-of-pocket and indirect costs were calculated for each norovirus event individually, as we were not able to rule out duplicative economic data within household clusters. We analyzed differences in out-of-pocket and indirect costs of norovirus events across demographic characteristics, severity of events, and level of healthcare utilization. To assess the severity of norovirus events, we calculated a rapid Vesikari score [21] and classified events as mild, moderate, or severe based on tertile across the cohort. We used generalized linear models to estimate the difference in mean costs and assess the statistical significance of differences across groups. Analyses were conducted in Stata statistical software, release 15 (StataCorp LLC, College Station, Texas, United States of America).

## Results

A total of 3,438 participants of all ages from 685 households were under surveillance during the study period. The population of PM had a higher proportion of children than SJ (S1 Table); children aged <5 years and those aged 5–17 years made up 15% and 30% of the population from PM, respectively, compared with 10.1% and 24% from SJ. Monthly household income was higher in PM, with 30% of households in PM earning in the highest income quartile (≥$1173.97), compared with 18% of households in SJ.

Among 2,128 AGE events with stool samples tested, we identified 330 (15.5%) laboratory-confirmed norovirus events among 266 individuals. Of these, 144 events occurred in PM and 186 in SJ (Table 1). The distribution of norovirus events was similar among male (52%) and female (48%) participants. Nearly half (49%) of all norovirus events occurred in children aged <5 years. The median duration of AGE symptoms among norovirus events was 3 days (IQR 2–4). The median rapid Vesikari score among norovirus events was 8 (IQR 7–9). Over half of (n = 174, 53%) norovirus events required a caregiver during the illness. While most ill persons did not seek medical attention, 75 (23%) norovirus events had an associated doctor’s visit or emergency room visit and 7 (2%) norovirus events required hospital admission. Health insurance utilization was reported for 42 (56%) norovirus events that received medical attention; 57% (n = 24) used public insurance (Seguro Integral de Salud), while 43% (n = 18) used private health insurance.

**Table 1:** Characteristics of laboratory-confirmed norovirus AGE events for Puerto Maldonado and San Jeronimo, Peru, 2012–2019.

Study population	Overall	Puerto Maldonado	San Jeronimo	p-value*
Norovirus-associated AGE events/total AGE events (%)	330/2128 (15.5%)	144/1114 (12.9)	186/1014 (18.3)	<0.01
**Demographics of ill persons**				
Female, n (%)	159 (48.2)	65 (44.2)	94 (50.5)	0.43
Age distribution, n (%)				
<5 years	162 (49.1)	84 (57.1)	78 (41.9)	0.05
5–17 years	59 (17.9)	25 (17)	34 (18.3)	0.90
18–44 years	55 (16.7)	19 (12.9)	36 (19.4)	0.55
v45–59 years	40 (12.1)	13 (8.8)	27 (14.5)	0.61
≥60 years	17 (5.2)	6 (4.1)	11 (5.9)	0.87
Average household members, median (IQR)	5 (4–7)	5 (4–7)	5 (4–7)	0.25
Cases with paid employment, n (%)	19 (5.8)	18 (12.2)	1 (0.5)	0.72
**Event characteristics**				
Rapid Vesikari score, median (IQR)	8 (7–9)	8 (7–9)	7 (7–9)	0.02
Number of days with symptoms, median (IQR)	3 (2 –4)	3 (1–5)	3 (2–4)	0.43
Level of healthcare utilization, n (%)^†^				
Received medical care^‡^	75 (22.7)	35 (23.8)	40 (21.5)	0.81
Non-medical care^§^	121 (36.7)	59 (40.1)	62 (33.3)	0.44
Utilized non-prescription medication/treatment	144 (43.6)	52 (35.4)	92 (49.5)	0.10
Hospitalized	7 (2.1)	4 (2.8)	3 (1.6)	0.46
Health insurance utilization, n (%)				
Comprehensive health system	24 (7.2)	10 (6.8)	14 (7.5)	0.95
Private insurance	18 (5.4)	12 (8.2)	6 (3.2)	0.69
**Caregivers**				
Needed caregiver, n (%)	174 (52.7)	65 (44.2)	109 (58.6)	0.07
Age of principal caregiver, median (IQR)	33 (27–40)	31.5 (26–40)	33 (27–40)	0.76
Caregivers with paid employment, n (%)	67 (38.5)	21 (32.3)	46 (42.2)	0.33

*T-test and Z-test for proportions were used for statistical comparisons among means across sites.

^†^Level of healthcare utilization was not mutually exclusive; i.e., participants may have sought multiple types of healthcare.

^‡^Medical care: Private physician, physician in health post, physician in hospital, emergency room.

^§^Non-medical care: Pharmacy, traditional healer, family/friends.

Among all norovirus events, 65% (210/330) incurred associated household costs. Among those that reported missed or impacted work or school time (indirect expenses), the median time lost was 0.75 days (IQR 0.5–1.0) among participants aged ≥3 years with norovirus events (n = 80) and 1 day (IQR 0.5–2.0) among caregivers (n = 51; Table 2). Money spent on prescription and non-prescription medications was the most commonly reported out-of-pocket cost (52% of events), followed by non-medical expenses (e.g., transportation, phone calls) incurred while seeking care (27%). Twenty-two cases reported expenses for diagnostic testing (more common in PM); when reported, testing was the most expensive of the defined categories (Table 2). Overall, expenses for medications accounted for 53% of out-of-pocket costs, followed by healthcare consultation (20%), non-medical expenses (15%), and diagnostic testing (12%).

**Table 2:** Costs associated with productivity loss and healthcare seeking among norovirus cases in Puerto Maldonado and San Jeronimo, Peru, 2012–2019.

Type of cost	Overall		Puerto Maldonado		San Jeronimo		
	n (%)	Median (IQR)	n (%)	Median (IQR)	n (%)	Median (IQR)	p-value*
Ill person time lost due to AGE (days)^†^	80 (43)	0.75 (0.5–1.0)	33 (51)	1.0 (0.5–1.25)	47 (39)	0.75 (0.5–1.0)	0.48
Caregiver time lost due to AGE (days)^‡^	51 (29)	1.0 (0.5–2.0)	38 (58)	1.0 (0.5–2.0)	13 (12)	1.0 (0.5–1.0)	<0.01
Money spent on healthcare consultation in USD ($)	45 (14)	$4.72 (3.56–17.79)	23 (16)	$6.45 (3.58–10.91)	22 (12)	$4.44 (3.08–21.02)	0.74
Money spent on diagnostic testing in USD ($)	22 (7)	$10.68 (4.35–19.61)	17 (12)	$10.83 (5.11–26.88)	5 (3)	$5.23 (4.02–18.02)	<0.01
Money spent on medication in USD ($)	170 (52)	$4.04 (1.44–8.58)	81 (56)	$4.10 (1.44–10.04)	89 (48)	$3.94 (1.48–7.85)	0.08
Money spent on non-medical expenses while seeking care in USD ($)	90 (27)	$1.18 (0.68–2.49)	62 (43)	$0.78 (0.70–1.94)	28 (15)	$1.55 (0.56–3.86)	0.07

*p-values represent comparisons between Puerto Maldonado and San Jeronimo, estimated by generalized linear model.

^†^Among cases ≥3 years of age.

^‡^Among cases with a reported caregiver.

The total cost to the household with a norovirus event had a median of $5.38 (IQR $2.15–$17.54), and a mean of $9.63 (SD $20.37). In PM, 74% of events incurred any out-of-pocket or indirect costs, with a median total cost of $7.82 (IQR $2.80–$27.36). In SJ, 55% of events had reported costs, with a median of $4.06 (IQR $1.47–$12.14). Norovirus events among children aged <5 years contributed 65% of norovirus-associated costs to the cohort; 73% of events in this age group incurred out-of-pocket or indirect costs, compared to 54% of events in ill persons aged ≥5 years.

Overall, out-of-pocket costs were incurred by 58% of all participants with norovirus events (187/330) with a median of $2.95 (IQR $1.04–$7.85) (Table 3), and a mean out-of-pocket cost of $3.98 (SD $9.27). On average, the out-of-pocket costs of a norovirus event were $4.35 higher among children aged <5 years compared to adults aged 18–44 years (p<0.01). Out-of-pocket costs increased with both AGE severity and healthcare utilization (Table 3). Notably, while half of all norovirus events occurred among children aged <5 years, this age group accounted for 71% (n = 75) of severe norovirus events, 72% (n = 55) of events with associated healthcare visits, and 86% (n = 6) hospitalizations.

**Table 3:** Comparison of out-of-pocket costs per laboratory-confirmed norovirus-associated AGE event, Peru, 2012–2019.

Characteristic of ill person or event	n (%)*	Median (IQR)^†^	Estimated difference in means	p-value^‡^
Overall	187 (58)	$2.95 (1.04–7.85)		
Puerto Maldonado	91 (63)	$2.97 (1.08–10.16)	$1.53	0.14
San Jeronimo	96 (52)	$2.80 (1.03–5.85)	Ref	
Monthly household income in USD ($) (quartiles)			-$0.11	0.83
≤$540.23	43 (56)	$2.67 (0.74–10.34)		
$540.24 –$785.49	43 (56)	$2.26 (1.04–5.28)		
$785.50 –$1173.96	46 (60)	$2.77 (0.95–5.90)		
≥$1173.97	42 (55)	$4.09 (1.33–9.08)		
Sex				
Male	104 (60)	$3.18 (1.41–9.12)	$1.60	0.12
Female	83 (53)	$2.66 (0.82–5.89)	Ref	
Age at time of event				
<5 years	112 (70)	$3.69 (1.77–10.81)	$4.35	<0.01
5–17 years	29 (49)	$1.51 (0.53–3.43)	$2.79	0.12
18–44 years	23 (43)	$1.17 (0.48–2.84)	Ref	
45–59 years	14 (36)	$3.47 (0.89–5.34)	$1.29	0.51
≥60 years	9 (53)	$3.05 (1.44–4.81)	$2.39	0.36
Rapid Vesikari score^§^				
Mild (5–7)	67 (45)	$1.80 (0.69–3.85)	$2.61	<0.01
Moderate (8)	45 (60)	$2.67 (0.88–4.35)		
Severe (9–14)	75 (71)	$5.8 (2.26–14.92)		
**Level of healthcare utilization** ^¶^				
Received medical care^||^				
Yes	70 (93)	$6.44 (2.43–17.59)	$9.91	<0.01
No	117 (46)	$2.15 (0.79–4.11)	Ref	
Received non-medical care**			
Yes	111 (93)	$3.05 (1.23–6.14)	$2.65	0.01
No	76 (36)	$2.43 (0.74–1.54)	Ref	
Utilized non-prescription medication/treatment				
Yes	24 (17)	$0.79 (0.41–2.02)	-$6.44	<0.01
No	163 (87)	$3.20 (1.33–9.03)	Ref	
Admitted to hospital				
Yes	7 (100)	$16.03 (3.52–7.74)	$8.24	0.02
No	171 (54)	$2.80 (1.01–6.46)	Ref	

*Number and percentage of cases in stratum reporting non-zero out-of-pocket costs.

^†^Cost distribution among those participants reporting non-zero out-of-pocket costs.

^‡^p-values estimated by generalized linear model.

^§^Rapid Vesikari scores were calculated for each event; severities were assigned after dividing scores into tertiles.

^¶^Level of healthcare utilization was not mutually exclusive; i.e., participants may have sought multiple types of healthcare.

^||^Medical care: Private physician, physician in health post, physician in hospital, emergency room.

**Non-medical care: Pharmacy, traditional healer, family/friends.

Indirect costs were reported for 28% of norovirus events overall (89/330) with a median of $12.58 (IQR $6.39–$25.16) (Table 4), and a mean indirect cost of $5.64 (SD $15.58). On average, indirect costs were $7.54 higher in PM than in SJ (p<0.01); the relationship between study site and indirect cost remained the same after adjusting for the ill person’s age. Increased severity, receipt of medical care, and hospitalization were associated with increased indirect costs, while use of non-prescription medication was associated with decreased indirect costs (Table 4).

**Table 4:** Comparison of indirect costs per laboratory-confirmed norovirus-associated AGE event, Peru, 2012–2019.

Characteristic of ill person or event	n (%)*	Median (IQR)^†^	Estimated difference in means	p-value^‡^
Overall	89 (28)	$12.58 (6.39–25.16)	NA	NA
Puerto Maldonado	60 (42)	$14.17 (6.32–29.16)	$7.54	<0.01
San Jeronimo	29 (16)	$9.59 (6.39–13.15)	Ref	
Monthly household income in USD ($) (quartiles)			$0.77	0.38
≤$540.23	20 (26)	$16.16 (6.34–30.73)		
$540.24 –$785.49	19 (25)	$11.00 (3.75–14.88)		
$785.50 –$1173.96	24 (31)	$12.80 (6.58–29.76)		
≥$1173.97	18 (23)	$12.16 (6.35–17.54)		
Sex				
Male	45 (26)	$12.91 (7.02–25.66)	$1.01	0.56
Female	44 (28)	$11.08 (6.23–19.63)	Ref	
Age at time of event				
<5 years	45 (28)	$14.17 (7.02–26.05)	$3.23	0.2
5–17 years	6 (10)	$14.02 (12.58–7.54)	-$2.36	0.43
18–44 years	18 (33)	$6.52 (3.72–14.88)	Ref	
45–59 years	13 (33)	$11.00 (6.51–12.91)	$2.28	0.5
≥60 years	7 (41)	$12.21 (9.44–43.98)	$7.39	0.09
Rapid Vesikari score^§^				
Mild (5–7)	21 (14)	$6.58 (3.69–12.58)	$5.15	<0.01
Moderate (8)	24 (32)	$11.68 (6.32–17.30)		
Severe (9–14)	44 (42)	$13.66 (7.33–29.16)		
**Level of healthcare utilization** ^¶^				
Received medical care^||^				
Yes	32 (43)	$14.02 (6.77–52.09)	$10.21	<0.01
No	57 (22)	$10.62 (6.35–17.54)	Ref	
Received non-medical care**				
Yes	37 (31)	$13.15 (6.58–25.40)	$2.51	0.17
No	52 (25)	$11.68 (6.29–23.84)	Ref	
Utilized non-prescription medication/treatment				
Yes	27 (19)	$9.59 (6.29–19.54)	-$5.33	<0.01
No	62 (33)	$13.15 (6.39–25.40)	Ref	
Admitted to hospital				
Yes	7 (100)	$71.01 (25.16–103.64)	$63.61	<0.01
No	81 (26)	$12.21 (6.39–19.54)	Ref	

*Number and percentage of cases in stratum reporting non-zero out-of-pocket costs.

^†^Cost distribution among those participants reporting non-zero out-of-pocket costs.

^‡^p-values estimated by generalized linear model.

^§^Rapid Vesikari scores were calculated for each event; severities were assigned after dividing scores into tertiles.

^¶^Level of healthcare utilization was not mutually exclusive; i.e., participants may have sought multiple types of healthcare.

^||^Medical care: Private physician, physician in health post, physician in hospital, emergency room.

**Non-medical care: Pharmacy, traditional healer, family/friends.

Productivity losses accounted for 59% of the total financial burden of norovirus events on households. Indirect costs were less frequently reported but were consistently higher than out-of-pocket costs for all age groups. Among ill persons of working age, indirect costs represented 77% of the total cost. On average, the cost of a given norovirus event represented 2.4% of the monthly household income for participants in the lowest quartile of household income (≤$540.23) and 1.1% for the highest quartile (≥$1173.97 in monthly income).

## Discussion

The design of this community cohort study, with intensive follow-up among participants of all ages who experienced AGE, allowed us to determine the out-of-pocket and indirect costs of norovirus events and estimate the financial impact of such events on households. The median cost of a norovirus event was $5, corresponding to 1–2% of monthly income among included participants. The burden of norovirus events was highest among children <5 years, representing 49% of all events and 65% of the total cost to households with norovirus AGE events in this study. Nearly a quarter of norovirus events required medical care. Both out-of-pocket and indirect costs were highest among those who were hospitalized. The financial burden of norovirus events was proportionately higher for households with a smaller income.

The mean total cost of a norovirus event estimated by our study ($9.63) is similar to the global average ($8.19, range: $2.89 in Pakistan–$18.89 in Kenya) of household expenses for children with medically attended norovirus reported in a previous study [22]. Similar to this previous study, the distribution of total cost per norovirus event in our study was skewed towards those events with higher costs, demonstrated by a lower median cost compared to the mean cost [22]. Studies assessing the economic impact of norovirus disease burden have estimated that globally, productivity losses represent 93% of the total economic burden among all age groups [12]. Comparatively, productivity losses represented a lower proportion of overall household costs (59%) in our study. This difference may be partially explained by the relatively low proportion of norovirus events among older adults in our study population.

Across age groups, children aged <5 years had the highest median out-of-pocket and indirect costs per norovirus event, which was largely explained by more severe illness and increased healthcare-seeking behaviors in this age group. An analysis of health service utilization from 2015 showed that in Peru children aged <4 years were among the groups most likely to seek outpatient care [23]. Although hospitalization is uncommon among norovirus cases, higher costs are reported when inpatient care is needed [22]. In our study, 6 of 7 norovirus-associated hospitalizations occurred in children aged <5 years. While our study focused on household costs, the highest total economic burden of norovirus on healthcare systems has also been attributed to this age group globally [12].

Although participants aged >60 years made up a small proportion of our study population resulting in small numbers and limited analysis in this age group, older adults had the highest proportion of events reporting indirect costs, and the second highest out-of-pocket costs after children aged <5 years. In global, model-based estimates, older adults have the highest indirect costs per norovirus illness [12]. Specifically, in the United States, people aged 45–65 years experiencing a norovirus event exhibited the largest associated indirect cost [13]. While older adults did not contribute a high proportion of total costs in our study population, norovirus events among this age group may have a larger economic impact in settings with older age distributions.

The results of this study are subject to some limitations. Participants were tested only for norovirus, so we could not eliminate the possibility that some AGE events or the severity of illness could be due to coinfections with other pathogens. The study was not designed to quantify expenses to the healthcare system through health insurance utilization or subsidized care, so this analysis is limited to out-of-pocket expenses and productivity losses incurred by participating households. For ill participants and caregivers aged ≥15 years who reported disruptions to daily activities but who did not report a monetary value, we assumed a daily or partial daily cost based on the per capita GDP in Peru. In doing so, we attempted to estimate the value to the household of their time, however, we recognize that these values do not represent true monetary costs. This approach may have also underestimated costs associated with illness among participants aged <15 years who may have assisted with family businesses or household labor. We did not assess the impact of household transmission and case clusters on household costs. Lastly, data were not collected during the same period from both sites; however, we adjusted for inflation to help reduce the impact on estimated costs.

Despite these limitations, this study provides economic estimates of the household costs of norovirus events based on data collected through almost seven total years of active surveillance conducted across two geographically distinct locations and among all ages, using a consistent case definition and methods. The study included laboratory-confirmed cases with milder symptoms that are typically not captured via hospital or clinic-based surveillance, thus providing unique information about the costs of norovirus AGE across all levels of severity. Unlike many studies that require diarrhea for recruitment and inclusion, the clinical case definition used in this study includes a vomiting-only presentation, which can be the only symptom of norovirus [1,24] and may contribute up to 20% of additional norovirus detections [17].

Most norovirus events identified in this study did not require medical care, supporting the need for community-based disease and economic burden studies. Previous norovirus studies in Peru have focused on children aged <5 years to characterize clinical symptoms of the disease [25,26], estimate incidence [25,27] and model the cost-effectiveness of introducing a childhood norovirus vaccination program using cost data from rotavirus studies [14]. The additional information provided by this study on the cost of a norovirus event to households may improve our understanding of the financial impacts of norovirus interventions. Our results suggest that a reduction in illness among children aged <5 years through interventions such as norovirus vaccines, which are progressing through development, may result in the greatest economic return. While the costs described in this paper suggest a substantial impact on households, these costs are only a fraction of the cost to society; cost-effectiveness studies for future norovirus vaccines should also incorporate costs to the healthcare system.

## Supporting information

S1 Table Demographic characteristics of study population by site. (DOCX)
