# Supplementary material for: Household economic costs of norovirus gastroenteritis in two community cohorts in Peru, 2012–2019
Source: PLOS Glob Public Health. 2024 Jul 10;4(7):e0002748. doi: 10.1371/journal.pgph.0002748 (PMC11236139; doi:10.1371/journal.pgph.0002748)
Supplement: S1 Table — (DOCX) [file pgph.0002748.s001.docx]

# **S1 Table. Demographic characteristics of study population by site**

| **Characteristic** | **Puerto**  **Maldonado** | **San Jeronimo** |  |
| --- | --- | --- | --- |
|  | **October 2012–August 2015** | **April 2015**–**April 2019** | **p-value** |
| Total population, n | 2173 | 1265 | NA |
| Total households, n | 431 | 254 | NA |
| Average household members, median (IQR) | 5 (4–6.5) | 5 (4–7) | 0.72* |
| Female sex, n (%) | 1182 (54.4) | 674 (53.3) | 0.53^†^ |
| Age distribution, n (%) |  |  |  |
| <5 years | 328 (15.1) | 128 (10.1) | <0.01^†^ |
| 5–17 years | 657 (30.2) | 308 (24.4) |  |
| 18–44 years | 793 (36.5) | 547 (43.2) |  |
| 45–59 years | 249 (12.8) | 172 (13.6) |  |
| ≥60 years | 116 (5.3) | 110 (8.7) |  |
| Monthly household income in USD ($), n (%) |  |  |  |
| <$540.24 | 177 (16.1) | 419 (28.8) | <0.01^†^ |
| $540.24–$785.49 | 270 (24.6) | 344 (23.6) |  |
| $785.50–$1173.96 | 244 (22.2) | 375 (25.8) |  |
| ≥$1173.97 | 330 (30.0) | 260 (17.9) |  |
| Not reported | 78 (7.1) | 57 (3.9) |  |
| IQR = interquartile range; USD = United States Dollars | | |  |
| *p-values represent comparisons between Puerto Maldonado and San Jeronimo, estimated using two-sample t-test | | | |
| ^†^p-values represent comparisons between Puerto Maldonado and San Jeronimo, estimated using chi-square tests | | | |
